# The Army Surgeon

**Published:** 1864-04

**Authors:** Baron Ambert


*The Army Surgeon.* By General Baron Ambert.

"There is not here below a more noble task. Those who fulfil it often purchase at the expense of their lives the modest glory which a world greedy of dazzling triumphs ignores. One might say of the military surgeon in the field what General Foy said of the captains of infantry: 'Strangers to the gratification of applause which the general officer enjoys, exempt from the passionate intoxication of the soldier, these martyrs to duty consume themselves in resignation.' Napoleon I. fully comprehended the importance of military surgery. He honored with an unlimited confidence the man in whom that profession was personified, the wise and devoted Larrey; he decreed to him a title of nobility. But beyond all this, on the rock of Saint Helena, he placed the name of Larrey in his last testament, with these words, which outvalue all titles, and surpass all eulogiums: 'C'est l'homme le plus vertueux que j'aie connu.'"

